# Comparison of Different Deodorizing Treatments on the Flavor of Paddy Field Carp, Analyzed by the E-Nose, E-Tongue and Gas Chromatography–Ion Mobility Spectrometry

**DOI:** 10.3390/foods13162623

**Published:** 2024-08-21

**Authors:** Chenying Fu, Yiming Zou, Yixiang Zhang, Mengxiang Liao, Duhuang Chen, Zebin Guo

**Affiliations:** 1 Engineering Research Centre of Fujian-Taiwan Special Marine Food Processing and Nutrition, Ministry of Education, Fuzhou 350002, China; fcy7600@163.com (C.F.); 15396063559@163.com (Y.Z.); zyx1027adu@163.com (Y.Z.); 2 College of Food Science, Fujian Agriculture and Forestry University, Fuzhou 350002, China; 3 Fujian Provincial Institute of Freshwater Fisheries, Fuzhou 350002, China; lmxiang2024@163.com (M.L.); chenduhuang@163.com (D.C.)

**Keywords:** paddy field carp, deodorization, gas chromatography–ion mobility spectrometry, electronic nose, electronic tongue

## Abstract

Changes in the flavor and taste profiles of Paddy Field Carp after deodorization with perilla juice (PJ), cooking wine (CW) and a mixture of the two (PJ-CW) were analyzed using the E-nose, E-tongue, gas chromatography–ion mobility spectrometry (GC-IMS), free amino acid analysis and taste nucleotide analysis. The E-nose and E-tongue revealed that deodorization reduced the content of sulfur-containing compounds, enhanced umami, bitterness, sourness and astringency, and decreased saltiness. PCA and OPLS-DA analysis successfully distinguished between the effects of the treatments. Free amino acids increased from 8777.67 to 11,125.98 mg/100 g and umami amino acids increased from 128.24 to 150.37 mg/100 g after PJ-CW deodorization (*p* < 0.05). Equivalent umami concentration (EUC) comparisons showed that PJ-CW treatment produced the greatest synergistic umami enhancement (to 3.15 g MSG equiv./100 g). GC-IMS detected 52 aroma compounds; PJ treatment produced the greatest diversity of aldehydes, including heptanal, nonanal, hexanal, 3-methylbutanal, (E)-2-heptenal and (E,E)-2,4-heptadienal. The total content of volatile flavor compounds was the highest after PJ-CW treatment, and the content of many characteristic flavor substances (3-hydroxy-2-butanone, benzaldehyde, 5-methyl-2(3H)-furanone) increased. These findings provided a theoretical basis for the further development of deodorization methods for Paddy Field Carp.

## 1. Introduction

The Chinese rice fish symbiotic system has a rich history spanning nearly a thousand years, and is globally recognized as an important agricultural heritage systems [1]. “Paddy Field Fish”, also known as “Rice Fish”, are raised in rice paddy fields and primarily rely on natural foods like rice flowers for growth and development. Diverse fish species can be integrated with rice cultivation, mainly including common carp (*Cyprinus carpio*) [2], crucian carp (*Carassius complex*), grass carp (*Ctenopharyngodon idella*), etc. The species studied here was “Paddy Field Carp” (*Cyprinus carpio*), which is popular with consumers for its tender and exceptionally nutritious flesh, and its richness in essential fatty acids, unsaturated fatty acids, amino acids and essential mineral elements [3]. However, past research on Paddy Field Carp mainly concentrated on quality characteristics and nutritional value, with only a few in-depth studies on the flavor characteristics and deodorization process.

Desirable flavor and taste are essential to the palatability of food, having a profound influence on its overall quality and shaping consumer preferences and acceptance [4]. In the production of fish-based processed foods, the presence of fishy odor is a major challenge [5]. As a kind of freshwater fish, Paddy Field Carp is susceptible to developing undesirable flavors such as earthy/muddy, fishy, and grassy flavors, which can be attributed to various sources and seriously affect its culinary appeal. Of these flavors, the earthy odor is primarily caused by the secondary metabolism of microorganisms such as planktonic algae, cyanobacteria and actinomycetes in fresh water, which produce earthy odors that are easily absorbed into the flesh of freshwater fish [6]. The main volatile components of fish muscle are aldehydes, alcohols and carbonyl compounds as their main volatile components, collectively accounting for >90% of the total odor volatile content [7]. The distinctive fishy or rancid odor of fish products is primarily attributed to compounds such as hexanal, (E,E)-2,4-decadienal, (E,E)-2,4-heptadienal, heptanal, nonanal, 1-penten-3-ol and 1-octen-3-ol [8]. There are four main methods for deodorizing aquatic products, namely physical, chemical, biological and composite deodorization [9,10]; however, sensory masking is the most commonly used approach. Sensory masking involves soaking, pickling and other processes to conceal fishy flavors with masking flavors; it has the advantages of cost-effectiveness and simplicity. Natural spices can mask unpleasant odors by incorporating them into food during the pickling and deodorization processes. In Asian countries, traditional spices and plant extracts such as ginger, cumin and thyme have been widely used to mask unpleasant odors and intensify the aroma of meat and diminish unpleasant odors, thereby improving the overall sensory quality and enjoyment of food [11]. Perilla (*Perilla frutescens L. Britt*.) is a traditional Chinese medicinal herb from the mint family that combines medicinal and culinary functions [12]. For culinary uses, perilla is usually used as an edible ingredient along with various seafoods from China, Japan and South Korea. It imparts a minty flavor to seafood dishes, which masks their unpleasant fishy odor [13]. Chinese cooking wine, also known as Huangjiu or Shaojiu, is a popular seasoning in traditional Chinese cuisine. It effectively eliminates fishy odor and pungent flavors when cooking meat, seafood and other ingredients, and can produce a rich aroma during the cooking process, making dishes more palatable [14].

In recent years, the electronic nose [15,16], electronic tongue [17] and gas chromatography–ion mobility spectrometry (GC-IMS) [18] methods have been widely used to characterize the volatile flavor compound profiles of aquatic products and analyze taste and flavor differences. Chen et al. [19] analyzed the volatile aroma components of wet-marinated fermented golden pomfret using electronic nose, GC-IMS and other techniques under different cooking methods. Through orthogonal partial least squares–discriminant analysis (OPLS-DA) and VIP analysis, 12 key flavor compounds were identified, including hexanal, isovaleraldehyde and (E)-2-dodecenal. Zhang et al. [20] used electronic nose and electronic tongue techniques to analyze the volatile flavor components and their production mechanisms of golden pompano (*Trachinotus blochii*) fillets under different drying methods. These results indicate that the combination of electronic nose, electronic tongue and GC-IMS technology can be well applied in the field of aquatic product flavor research, and the differences between flavor substances can be compared by combining OPLS-DA and VIP analyses. However, the combination of E-nose, E-tongue and GC-IMS has not been used to analyze the flavor and taste characteristics of deodorized Paddy Field Carp.

Different deodorization methods have different effects on fish and on the flavor of the product. Therefore, in this study, the E-nose and E-tongue systems were used to distinguish the flavor and taste differences in Paddy Field Carp after three deodorization treatments. Volatile compounds in deodorized fish muscle were analyzed by GC-IMS to identify the flavor-active constituents and generate a flavor fingerprint. Free amino acids, taste activity values and flavor nucleotides were analyzed and subjected to principal component analysis (PCA) and orthogonal partial least squares–discriminant analysis (OPLS-DA) to investigate taste changes before and after deodorization. The integration of different analytical techniques allows for a multidimensional assessment of volatile compounds, taste activity values and flavor nucleotides, providing a more nuanced understanding of the flavor profile. The use of traditional spices and plant extracts for deodorization not only modifies Paddy Field Carp’s flavor characteristics but also enhances consumer acceptance and preference, thereby addressing a specific challenge in the processing of freshwater fish. The findings provided an overview of volatile compound composition and explained the underlying mechanisms that govern variations in the fishy flavor of Paddy Field Carp. The aim was to improve the quality, optimize deodorization methods, and establish a theoretical foundation for the future development and practical application of Paddy Field Carp products.

## 2. Materials and Methods

### 2.1. Sample Preparation

All Paddy Field Carp samples were provided by Fujian Freshwater Fisheries Research Institute (Cangshan District, Fuzhou, Fujian, China) and were raised at the Lanfeng Paddy Field Fish Aquaculture Farmers’ Professional Cooperatives in Wuyishan City, Fujian Province. Approximately 300 fish were released into experimental field, and the fish fry were soaked in 3% salt water for 5 min before being placed in the field. The breeding cycle of fish is 60 days, and the fish fry feed on rice pollen, rice husks, field duckweed, weeds, aquatic planktonic animals, plants and pests. Paddy Field Carp muscle samples (average weight (90.38 ± 5.81) g) were selected for deodorization treatment and stored in a −80 °C freezer. The fish was thawed at room temperature before use, then cut into small pieces (3 × 3 × 1 cm).

The fish samples were divided into four treatment groups, i.e., Blank (B), which was not deodorized; perilla juice (PJ), treated with perilla juice (5% aqueous solution, stored at 4 °C); cooking wine (CW), treated with cooking wine (5% aqueous solution, stored at 4 °C); and combined perilla juice–cooking wine (PJ-CW), perilla juice mixed with cooking wine (1:3 ratio), as a 5% aqueous solution, stored at 4 °C. The fish muscle samples were minced with a meat grinder and soaked in the treatment solutions at room temperature for 30 min. Perilla juice (perilla leaf distilled extract, purity 100%) was obtained from Zhejiang Shizi Biotechnology Co., Ltd. (Jiangshan, Zhejiang, China); Cooking wine was obtained from Fujian Laojiu Liquor Industry Co., Ltd. (Fuzhou, Fujian, China). In each individual experiment (E-nose, E-tongue, GC-IMS, free amino acids and nucleotides), three fish of similar size in each treatment group were used for testing (groups B, PJ, CW and PJ-CW).

### 2.2. Electronic Nose Analysis

The flavor profiles of Paddy Field Carp samples were determined as described previously [21], with some modifications, using a PEN3 E-nose system (Airsense Analytics GmbH, Schwerin, Germany). Three fish of similar size were selected for parallel experiments in each treatment group. Samples (2.00 g) were placed in a 20 mL headspace vial and left to stand at room temperature (25 °C) for 30 min, then analyzed with the following settings: gas flow rate 400 mL/min, cleaning time 180 s and measurement time 180 s. The analysis was performed in triplicate and radar charts were used to display the mean values obtained.

### 2.3. Electronic Tongue Analysis

The E-tongue analysis was performed as described previously [22], with some modifications, using an SA-402B E-tongue system (Beijing Yingsheng Hengtai Technology Co., Ltd. Beijing, China). Three fish of similar size were selected for parallel experiments in each treatment group. The sample was taken out from a −80 °C refrigerator and thawed in a 40 °C water bath until the center temperature reached 40 °C. Four fish samples (5 g) were thoroughly mixed with 25 mL distilled water at 40 °C, respectively, then centrifuged at 3000× *g* at 4 °C for 10 min. The supernatant was then filtered through medium-speed qualitative filter paper to prepare it for the E-tongue analysis. Artificial saliva, containing KCl (30 mM), and tartaric acid (0.3 mM) were used as the reference solution to simulate human oral saliva. Each sample was analyzed in triplicate, then the data were processed using the instrument’s integrated database and software. Radar and bubble charts were generated to visualize the results. The E-tongue was able to detect alterations in membrane potential resulting from electrostatic or hydrophobic interactions between various flavor compounds and artificial lipid membranes; this enabled the evaluation of the five fundamental taste attributes as well as the astringency [23].

### 2.4. GC-IMS Analysis

The flavor profile analysis was performed using a GC-IMS (FlavorSpec^®^) system from Gesellschaft für analytische Sensorsysteme mbH (GAS, Dortmund, Germany), as described by Xiao et al. [24] with minor revisions. The instrument was equipped with an MXT-5 capillary column (15 m × 0.53 mm × 1.0 μm; Restek, Westport, CT, USA). Three fish of similar size were selected for parallel experiments in each treatment group. Briefly, the sample (2 g) was sealed in a 20 mL headspace vial, heated with magnetic stirring at 60 °C for 15 min, then an aliquot (500 μL) of the headspace vapor was removed and injected into the GC in splitless mode. The injector temperature was 85 °C.

The GC-IMS analysis was performed at constant temperature (60 °C) for 30 min, with nitrogen as the drift gas at 75 mL/min. Ionization mode: positive ion mode. IMS temperature: 45 °C. The carrier gas (99.999% purity nitrogen) was programmed with a variable flow rate, initially 2 mL/min, increased to 10 mL/min for 8 min, then to 100 mL/min for 10 min. Each sample was analyzed in triplicate. Retention indices (RIs) were determined using a series of C_4_–C_9_ n-alkanes as external standards. Volatile compound identification was accomplished by comparing their retention indices (RIs) with those of known standards in the GAS GC-IMS library. Qualitative analysis of flavor compounds was performed using Laboratory Analytical Viewer (LAV) software (Version 0.4.03) combined with the GC-IMS Library Search tool, the NIST 2020 database (National Institute of Standards and Technology, Gaithersburg, MD, USA) and the Information Management System Database.

### 2.5. Free Amino Acid (FAA) Analysis

The FAA content was determined using the AQC (6-aminoquinolyl-N-hydroxy-succinimidyl carbamate) derivatization method as described by Zhou et al. [25] with slight adjustments. Three fish of similar size were selected for parallel experiments in each treatment group. Sample (~0.20 g) was hydrolyzed with hydrochloric acid (10 mL, 6 mol/L) at 110 ± 1 °C for 22 h, cooled to ambient temperature and the volume adjusted to 10 mL with water.

The hydrolyzed solution was centrifuged and filtered through a 0.22 μm microporous membrane. An aliquot (10.0 μL) was combined with AccQ-Fluor borate buffer (70.0 μL, pH 8.8); then, AccQ-Fluor reagent (20.0 μL, 3 g/L) was added. The mixture was vortexed and incubated at 55 °C for 10 min, then cooled to room temperature prior to analysis.

### 2.6. Taste Activity Value (TAV) Analysis

To assess the influence of taste-active substances on Paddy Field Carp, their taste activity values (TAVs) were calculated using Equation (1) [26]:

TAC = C1/C2(1)

where C1 (mg/100 g) is the concentration of an individual compound in the sample and C2 (mg/100 g) is the detection threshold of that compound. A substance is considered to significantly influence the taste profile when its TAV is >1.

### 2.7. Taste Nucleotide Analysis

Taste nucleotides ware determined by liquid chromatography, following the protocols in China National Standard: GB 5413.40-2016 [27]. Three fish of similar size were selected for parallel experiments in each treatment group. Sample preparation: Mix 5.00 g of sample with 50 mL of ultrapure water for 3 min, sonicate at room temperature for 40 min, heat at 100 °C for 15 min, centrifuge at 4 °C for 15 min at a speed of 4000 r/min, retain the supernatant, filter with a 0.45 μm membrane, and measure the filtrate on the machine.

Operating conditions: HPLC-MS/MS analysis was performed on a Vanquish UHPLC system connected to a TSQ Quantis triple quadrupole mass spectrometer using a Thermo Fisher-C18 (150 mm × 2.1 mm × 2.7 μm) chromatography column. The mobile phase was set to 0.1% formic acid water (A) and acetonitrile (B) for gradient elution, with a flow rate of 0.3 mL/min and an injection volume of 5 μL. The MS detection parameter is the ion source: H-ESI. The scanning method is negative ion scanning.

### 2.8. Equivalent Umami Concentration (EUC)

The influence of free amino acids and nucleotides on umami taste perception was evaluated by calculating the equivalent umami concentration (EUC), using Equation (2) [28]:

EUC = ∑ai × bi + 1218 (∑ai × bi) (∑aj × bj) (2)

The EUC is expressed as grams of monosodium glutamate (MSG) equivalent/100 g; ai and bi represent the quantity of the umami amino acid (L-Glu) and its umami coefficient, respectively, relative to MSG (glutamate is 1.0). The terms aj and bj represent the quantity of nucleotides, such as AMP (adenosine monophosphate) or IMP (disodium inosine-5′-monophosphate), and their umami coefficients (IMP 1, AMP, 0.18), respectively; 1218 is the synergy constant. The EUC values were applied to quantify the combined effect on perceived umami intensity of nucleotides and L-Glu.

### 2.9. Statistical Analysis

In each experiment (E-nose, E-tongue, GC-IMS, free amino acids and nucleotides), three fish were tested in each of the four treatment groups. Data of free amino acids and nucleotides were expressed as mean ± standard deviation and were used in SPSS 25.0 software to perform differential analysis by ANOVA (SPSS Inc., Chicago, IL, USA). The tables were generated using Excel 2016 software (Microsoft Corporation, Redmond, WA, USA).

The response values of electronic nose and electronic tongue sensors are represented by radar charts and bubble charts. The radar charts and bubble charts were generated with Origin 2023 (OriginLab Co., Northampton, MA, USA). Simca 14.1 (Umetrics AB, Umea, Vasterbotten, Sweden) was used for principal component analysis (PCA) with scaling-type UV and orthogonal partial least squares–discriminant analysis (OPLS-DA). OPLS-DA, PCA, radar chart and bubble chart analyses on the sensor response values of the electronic nose and electronic tongue were used to characterize the differences in odor and taste among carp samples.

The changes in the content of volatile flavor compounds before and after deodorization are represented using a histogram generated by GraphPad Prism 9.5.1 (GraphPad Software, Inc., San Diego, CA, USA).

## 3. Results and Discussion

### 3.1. Electronic Nose Analysis

#### 3.1.1. Flavor Profile

**Figure 1:**
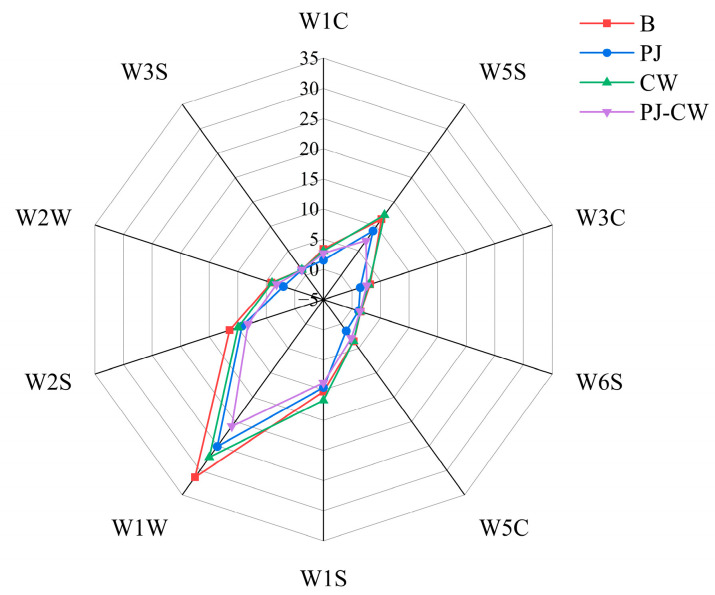
E-nose radar plot of Paddy Field Carp with different deodorization treatments. Note: B: blank group; PJ: perilla juice; CW: cooking wine; PJ-CW: perilla juice and cooking wine. The scale value (from −5 to 35) represents the response value of the sensor, which is the ratio of the conductivity G of the sample gas passing through the sensor to the conductivity Go of the standard gas filtered by activated carbon passing through the sensor, i.e., G/Go.

The E-nose simulates the sensory mechanisms of the mammalian olfactory system, and can recognize both simple and complex odors [29]. Due to the strong response of each sensor to a certain type of characteristic gas, we can determine which type of volatile gas is the main one in the sample analysis process. The flavor characteristics of the four deodorization treatments (Blank—B; perilla juice—PJ; cooking wine—CW; and perilla juice and cooking wine—PJ-CW) were determined using the E-nose through its 10 sensors (Table 1). The response intensities of the W1W sensor (sulfides), W1S (methyl), W2S (alcohols/aldehydes/ketones) and W5S (nitrogen oxides) were significantly lower after deodorization, whereas only minor changes were observed in the other six sensors (Figure 1). Deodorization particularly reduced the content of inorganic and organic sulfides, which may be related to the modification of protein sulfhydryl groups [30]; nitrogen and sulfur compounds are associated with fishy and egg odors [31]. The presence of sulfides in fish muscle is associated with the biochemical reactions occurring during spoilage; bacteria break down proteins and sulfur-containing amino acids in fish muscle, producing compounds with an unpleasant odor, such as hydrogen sulfide (H_2_S). The E-nose was clearly able to distinguish odor differences before and after deodorization.

#### 3.1.2. Orthogonal Partial Least Squares–Discriminant Analysis (OPLS-DA)

**Figure 2:**
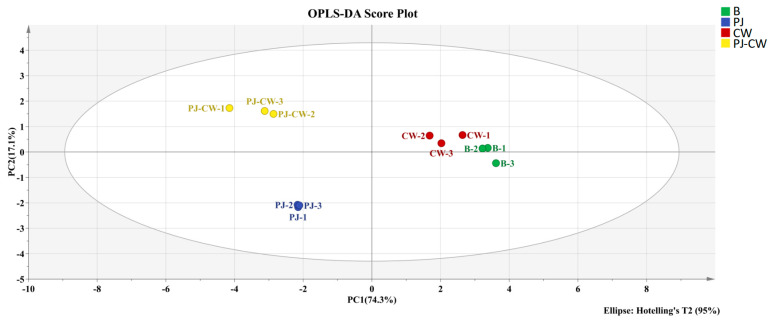
E-nose OPLS-DA score plot of Paddy Field Carp with different deodorization treatments (R^2^X = 0.995; R^2^Y = 0.979; Q^2^ = 0.957). Note: B: blank group; PJ: perilla juice; CW: cooking wine; PJ-CW: perilla juice and cooking wine.

**Figure 3:**
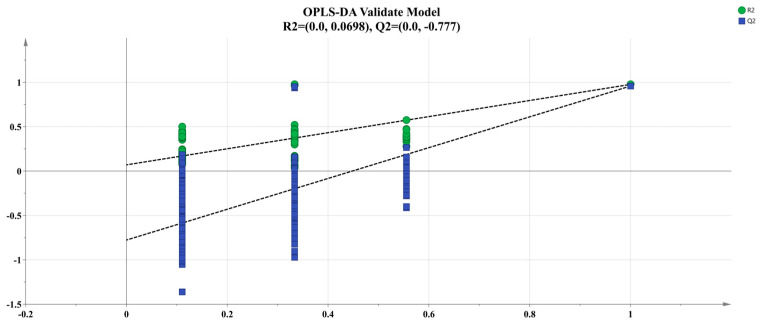
OPLS-DA permutation test.

The OPLS-DA analysis of the sensor response values obtained from the electronic nose testing of four samples was used in order to differentiate the odor differences between the four samples (Figure 2 and Figure 3). OPLS-DA is an advanced version of the PLS-DA approach, employing separate predictive and orthogonal factors to highlight differences among and within sample groups [32]. This method eliminates irrelevant independent variables that do not contribute to differentiation, thereby identifying the distinctive variable characteristics of the sample [33]. Using the response values of four sample electronic nose sensors as Y variables for OPLS-DA modeling design, the explanatory power of the model is expressed as the R^2^X and R^2^Y values, and the predictive ability of the model is expressed as Q^2^; R^2^ and Q^2^ values closer to 1.0 indicate a good fit of the model to the data [34]. The response values of four sample electronic noses were analyzed using the OPLS-DA model (Figure 2), where R^2^X = 0.995, R^2^Y = 0.979 and Q^2^ = 0.957, which are all close to 1, indicating that the model has good explanatory and predictive power. We performed 200 cross-permutation tests on the model to test the reliability of the OPLS-DA model (Figure 3). The horizontal axis in the figure represents the retention of the sample, and the point at 1.0 represents the R^2^ and Q^2^ of the original model. After verification, both R^2^ and Q^2^ are less than the value with a retention of 1.0, and the intercept between the regression line of model Q^2^ and the *x*-axis is negative, indicating that the model is not overfitting and the constructed model is reliable.

**Figure 4:**
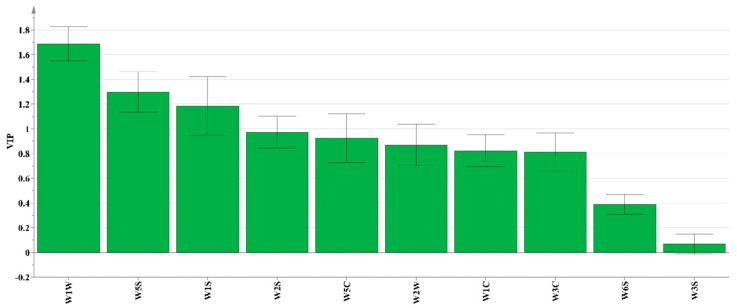
E-nose VIP value of Paddy Field Carp with different deodorization treatments.

The total variance detected by the PCA analysis contributed by principal component PC1 and PC2 was 91.4%, i.e., it effectively differentiated the flavor profiles of the four deodorization treatments. The PJ-CW and PJ groups were clustered, but well separated on the positive and negative sides of PC2, respectively; similarly, the B and CW groups were clustered, but well separated in the first and fourth quadrants of PC1. The variable importance in projection (VIP) value is commonly used to identify the key variables in OPLS-DA models; the higher the VIP value (VIP > 1 indicates a significant variable), the greater the contribution of the substance to differentiation between samples [35]. Three of the E-nose sensors accounted for significant contributions to sample differentiation (W1W, sensitive to sulfides; W5S, sensitive to nitrogen oxides; W1S, sensitive to methyl), wherein W1W had the highest VIP value, followed by W5S and W2S, indicating that sulfides, nitrogen oxides and methyl compounds differentiated the deodorization treatments (Figure 4).

### 3.2. Electronic Tongue

#### 3.2.1. Flavor Profile

**Figure 5:**
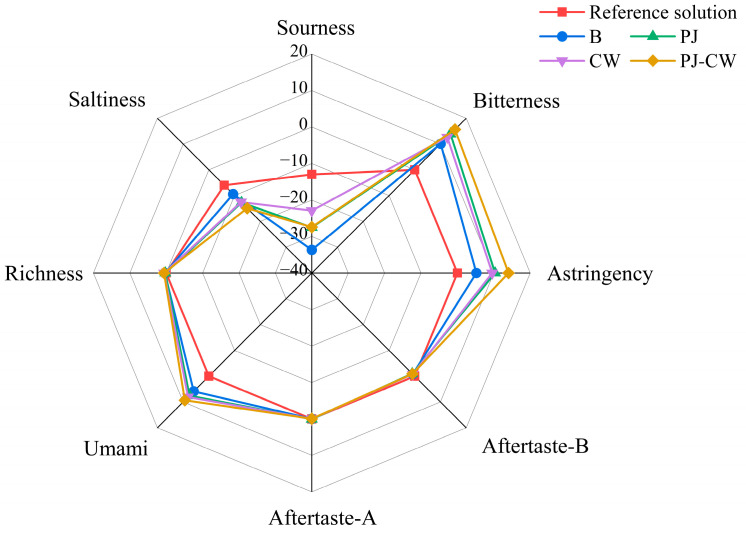
E-tongue radar plot of Paddy Field Carp with different deodorization treatments. Note: B: blank group; PJ: perilla juice; CW: cooking wine; PJ-CW: perilla juice and cooking wine. The scale value (from −40 to 20) indicates that the response value of the sensor is equivalent to the level of taste value.

The electronic tongue can distinguish and categorize various taste substances using the responses from its sensors, simulating the sensory mechanisms of the mammalian taste system, and is a promising qualitative analysis technology [36]. The electronic tongue is composed of multiple taste sensors, each of which has different responses to different chemical components. They generate electrical signals based on the chemical composition in the sample and convert them into digital signals. These sensor arrays simulate the taste buds on the human tongue and can distinguish among eight basic taste signals in the sample: sourness, bitterness, astringency, umami, saltiness, richness (umami aftertaste), bitter aftertaste, and astringency aftertaste (Figure 5).

Artificial saliva is composed of potassium chloride and tartaric acid, and its E-tongue response is defined as the tasteless or zero point. The tasteless point for the sourness was −13, the salty taste was −6, while the other six taste signals, it was 0. E-tongue responses of a sample equal to, or one or more responses above, the zero point indicated the presence of more characteristic taste components in the sample, whereas a point lower than the zero point indicated relatively less [37]. The basic taste profile of carp changed after the different deodorization treatments (*p* < 0.05), mainly reflected as differences in umami, saltiness, sourness, astringency and bitterness, so these parameters effectively differentiated among the taste profiles of the four treatments. The E-tongue responses for sourness, saltiness, aftertaste-B and aftertaste-A were below the zero point, indicating that there is no obvious taste or relatively weak intensity.

**Figure 6:**
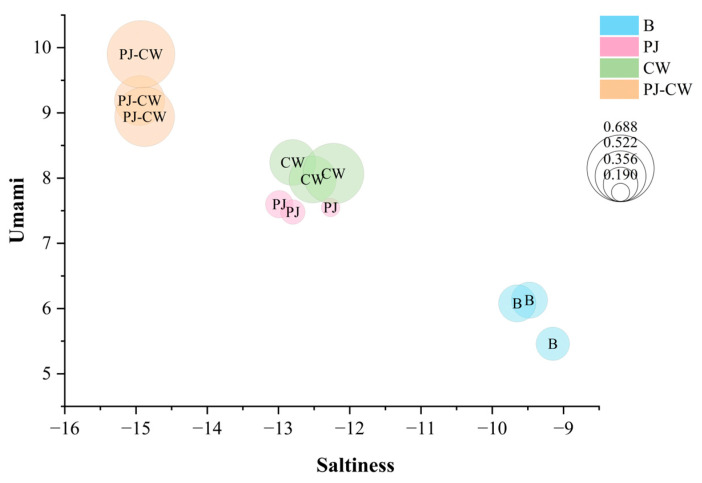
Saltiness, umami and richness bubble chart of Paddy Field Carp with different deodorization methods. Note: B: blank group; PJ: perilla juice; CW: cooking wine; PJ-CW: perilla juice and cooking wine.

To more accurately elucidate the effects of taste changes, further analysis was conducted on the E-tongue data through bubble charts. Bubble charts analyze relationships between variables; each bubble represents a single data point, the values for which are indicated by horizontal position, vertical position and bubble size. A bubble chart for the deodorization treatments of Paddy Field Carp, showing saltiness, umami and richness, was generated (Figure 6) and shows the high repeatability of the three replicates of each sample. The umami intensity (*Y*-axis) markedly increased after the three deodorization treatments, with PJ-CW strengthening umami intensity the most, followed by the CW and PJ treatments, respectively. The bubble size is directly proportional to the richness value; the three deodorization methods effectively reduced the saltiness (*X*-axis) of the fish muscle and maintained its richness of taste. Cooking wine removes the fishy smell and enhances the umami intensity of fish meat [38]; the alcohol in cooking wine can dissolve and thereby remove fishy substances such as trimethylamine and volatile sulfides from fish, in agreement with the E-nose findings.

**Figure 7:**
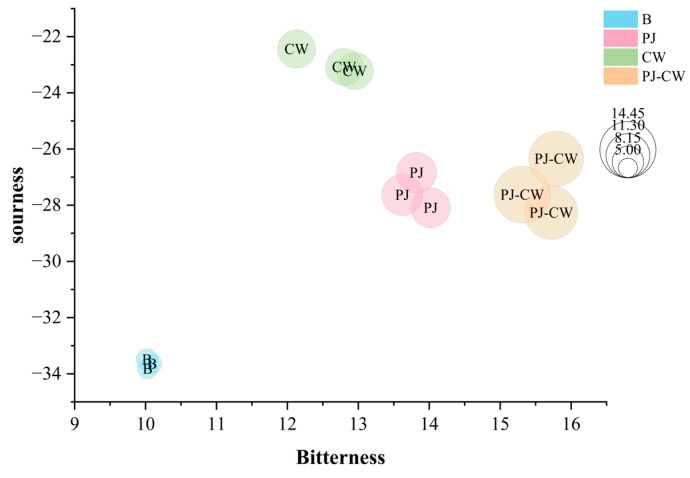
Bitterness, sourness and astringency bubble chart of Paddy Field Carp with different deodorization methods. Note: B: blank group; PJ: perilla juice; CW: cooking wine; PJ-CW: perilla juice and cooking wine.

Bitterness, sourness and astringency were also significantly different before and after deodorization (Figure 7), and all three taste attributes increased after the deodorization treatments. The sourness intensity was the highest after CW deodorization, whereas the bitterness and astringency were the highest after PJ-CW deodorization. The alkaloids, terpenoids and phenolic compounds in perilla juice increased the bitterness and astringency of fish muscle [39].

#### 3.2.2. Principal Component Analysis (PCA)

**Figure 8:**
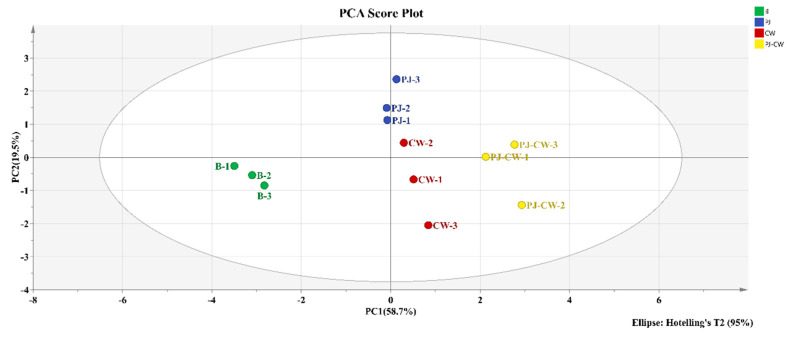
E-tongue PCA score plot of Paddy Field Carp with different deodorization treatments. Note: B: blank group; PJ: perilla juice; CW: cooking wine; PJ-CW: perilla juice and cooking wine.

PCA of the observed taste characteristics was used to detect any anomalous data points and to reveal the relationships between samples [40]. The PCA results of the four treatment carp samples, based on the response values of the E-tongue sensor, are presented in Figure 8 in order to differentiate the taste differences between the four samples. Different colored areas in the PCA plots represent the overall taste distribution of each deodorization treatment. The accumulative contribution rate of the two principal components is 78.2%, suggesting that these two principal components can reflect the overall odor information of carp. PC1 accounts for 58.7% of the total contribution rate, while PC2 accounts for 19.5% of the total contribution rate. PC1 represents 58.7% and PC2, 19.5% of the total variance; therefore, PC1 is the main principal component used to differentiate the four treatments, which are clearly separated over the four different areas of the PCA plot. This result is consistent with the results of significantly distinguishing the four treatments in the radar image (Figure 5).

### 3.3. Amino Acid Content

Amino acid analysis detected 18 amino acids. According to the different chemical properties of the side chains (R groups), they can be divided into ten bitter, ten sweet, two umami, two sour and six essential amino acids (Table 2). The amino acid content increased after all three deodorization treatments (*p* < 0.05), i.e., Blank, 8777.67; PJ, 9545.51; CW, 9664.19; and PJ-CW, 11,125.98 mg/100 g. Deodorization also increased the essential amino acid content (Glu and Gln). The amino acids that influenced the taste of Paddy Field Carp were from the categories of sweet, bitter, umami and sour amino acids. Umami was an essential contributor to the taste profile of fish. Research by Cao et al. [41] showed that the main amino acids in tilapia fillets were the umami amino acids, including Glu and Asp. The PJ and PJ-CW deodorization methods both significantly increased the EUC to 150.37 and 135.25 mg MSG equiv./100 g, respectively, in agreement with the E-tongue findings. The amino acids and peptides present in PJ, but not in CW, may interact with proteins in fish muscle to enhance their umami intensity. Sweet and bitter amino acids predominated after deodorization, and the highest content of these amino acids was 7913.34 and 8462.95 mg/100 g, respectively, after PJ-CW treatment. Essential amino acids make an important contribution to the nutritional value and taste of fish meat, and they are also essential nutrients for fish growth; all three deodorization treatments increased the essential amino acid content.

Taste activity value (TAV) is a commonly used measure of the flavor intensity of food and the contribution of individual components to the overall flavor [42]. If the TAV value is >1, the substance is considered to contribute significantly to the taste profile, so the determination of TAVs can identify the primary taste-active substances. The TAV values of amino acids after all the deodorization treatments were >1, and the amino acids with the highest TAVs were Lys and Arg, indicating that all free amino acids contributed to the taste of carp.

### 3.4. Taste Nucleotide

**Figure 9:**
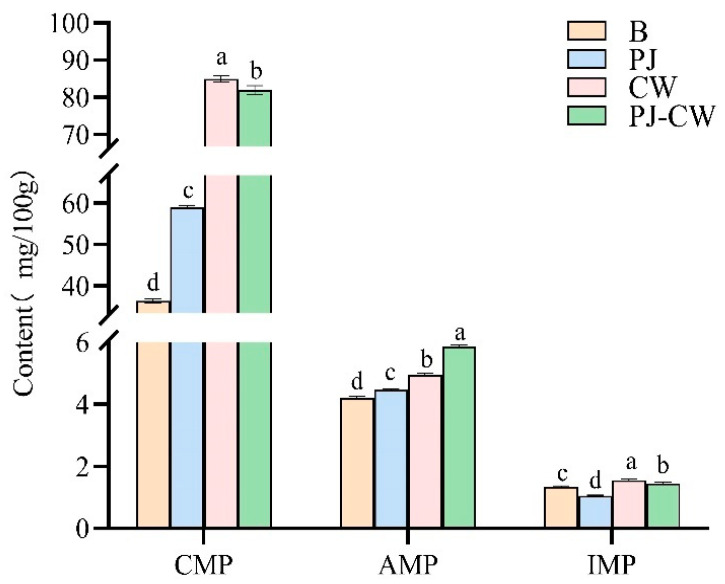
Nucleotide content of Paddy Field Carp with different deodorization methods. Note: B: blank group; PJ: perilla juice; CW: cooking wine; PJ-CW: perilla juice and cooking wine. “AMP” is 5′-adenosine monophosphate; “GMP” is 5′-guanosine monophosphate and “CMP” is 5′-cytidine monophosphate. Different letters in the figure indicate significant differences (*p* < 0.05).

The primary taste components in carp muscle are free amino acids and nucleotides, which contributed to its taste profile, either directly or through associated effects. The taste nucleotides, namely 5′-inosine monophosphate (IMP), 5′-adenosine monophosphate (AMP), 5′-guanosine monophosphate (GMP) and 5′-cytidine monophosphate (CMP), are major contributors to the umami taste of aquatic products [42]. The most abundant nucleotides in carp were IMP, AMP and CMP (Figure 9), with CMP the most abundant in the blank and the three deodorization treatments, followed by AMP and IMP, i.e., CMP made the largest contribution to the distinctive taste of carp, both before and after deodorization. CMP is a flavor enhancer, particularly for the taste profile of meat and seafood dishes. When added to fish meat, it contributes to its umami intensity. These nucleotides interacted synergistically with umami amino acids, resulting in a substantial enhancement of the umami intensity and taste profile of Paddy Field Carp.

The order of umami intensity enhancement capacity is GMP > IMP > AMP [43]. The nucleotide content of Paddy Field Carp before and after deodorization is summarized in Table 3. After the deodorization treatments, the content of three flavor nucleotides in fish muscle markedly increased (*p* < 0.05). EUC is a key taste evaluation metric, reflecting the umami intensity of aquatic products, and highlighting the synergistic interactions between umami-enhancing nucleotides and amino acids. The EUC values of the Blank, PJ, CW and PJ-CW were 2.64, 2.32, 3.07 and 3.15 gMSG/100 g, respectively, with the highest TAV in all four samples for AMP, i.e., the PJ-CW treatment produced the highest umami intensity.

### 3.5. Volatile Components Analysis

#### 3.5.1. GC-IMS Two-Dimensional Analysis

**Figure 10:**
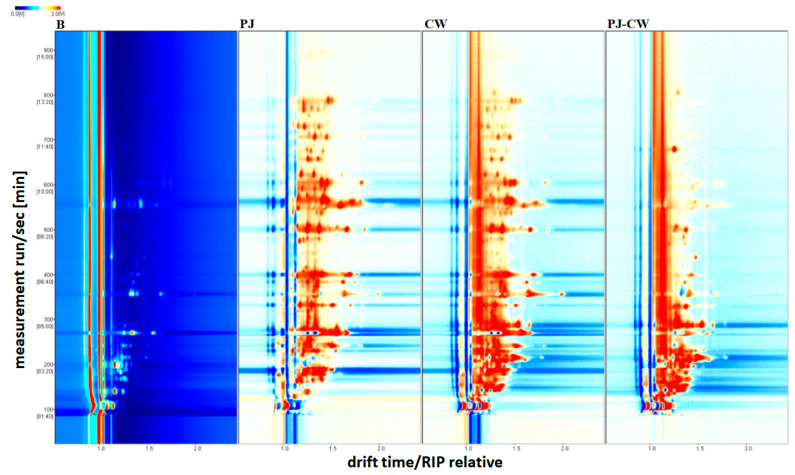
Comparison of volatile substances in Paddy Field Carp with different deodorization methods. Note: B: blank group; PJ: perilla juice; CW: cooking wine; PJ-CW: perilla juice and cooking wine. The B group serving as the reference, the two-dimensional difference spectra of the other three groups were obtained by deducting the reference, where the background turned white [46]. The red vertical lines represent the reaction ion peak (RIP), and each point on both sides of them represents a volatile substance. The spots with different colors represent different concentrations of each volatile organic compound. The blue area indicates that the concentration in the sample is lower than the B group, while the red area indicates that the concentration is higher than the B group.

In recent years, GC-IMS analysis technology has been widely used as a fast, non-destructive, and highly sensitive detection technique for the determination of volatile flavor compounds in aquatic products [19,44,45]. Figure 10 shows a two-dimensional differential map of GC-IMS spectra obtained from fish muscle samples. It was clear that there were marked differences between the Blank and the three deodorized samples. The spectra of the deodorized samples had many red spots and few blue spots, indicating that deodorization increased the abundance of volatile flavor compounds. Wang et al. [38] found that adding perilla to traditional Chinese fish dishes can increase the volatile flavor compounds in fish. Xiao et al. [24] used GC-IMS analysis technology to study the effects of four different cleaning processes on the volatile flavor compounds of grass carp. The results showed that weak alkaline substances can effectively reduce the fishy substances in grass carp.

#### 3.5.2. Fingerprint Analysis of VOCs in Paddy Field Carp

**Figure 11:**
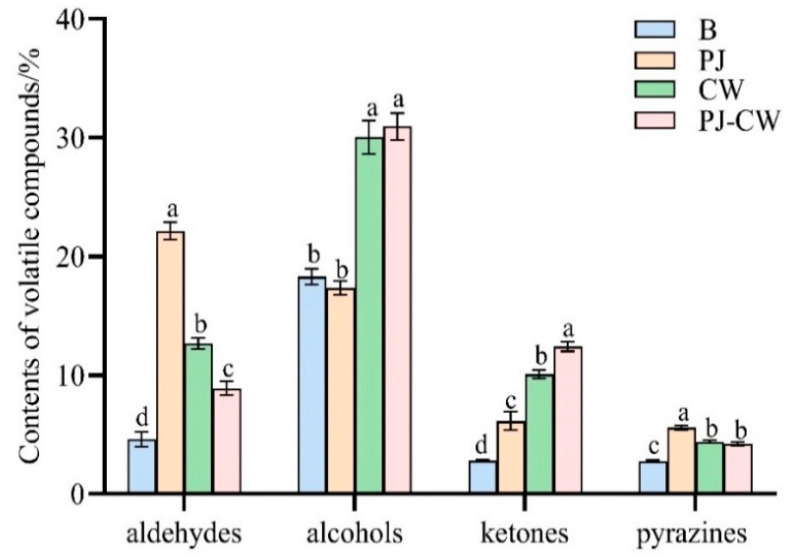
Histogram of the content of some volatile substances in Paddy Field Carp with different deodorization methods. Note: B: blank group; PJ: perilla juice; CW: cooking wine; PJ-CW: perilla juice and cooking wine. Different letters in the figure indicate significant differences (*p* < 0.05).

Alcohols (4.61%) and aldehydes (18.30%) were the most abundant flavor compound classes before deodorization, whereas ketones (2.81%), pyrazines (2.78%) and esters (0.11%) were present in relatively low concentrations (Figure 11). After PJ deodorization, aldehydes, ketones and pyrazines were significantly more abundant, whereas alcohols were slightly less abundant. Both CW and PJ-CW deodorization increased the abundance of aldehydes, alcohols, ketones and pyrazines.

**Figure 12:**
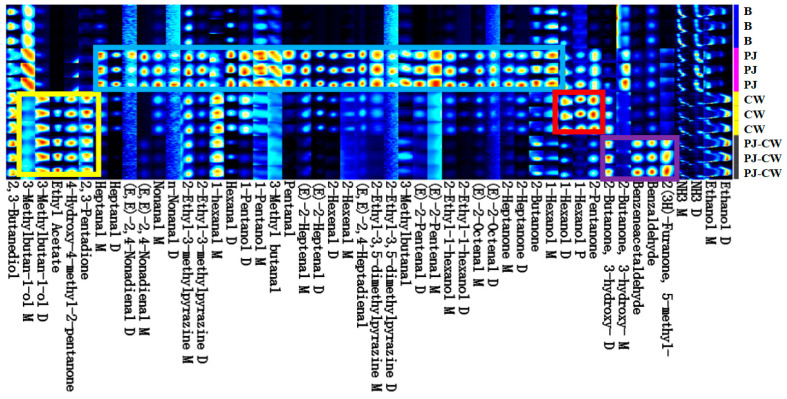
GC-IMS volatile substance fingerprint. Note: The areas enclosed by blue, yellow, purple and red represent the characteristic flavor substances of Paddy Field Carp under different deodorization methods, respectively. B: blank group; PJ: perilla juice; CW: cooking wine; PJ-CW: perilla juice and cooking wine. “M” is Monomers; “D” is Dimers; “M” and “D” are actually one substance, with the same retention time but different migration times.

The fishy smell of fish meat arises from various volatile compounds, with a complex composition. The unpleasant odor similar to metal or soil in freshwater fish is mainly produced by volatile substances such as alcohols, aldehydes and ketones, and are easily influenced by their own growth conditions and external environment [24]. To more intuitively compare the differences in volatile flavor compound compositional differences after treatment with the three deodorization methods, their GC-IMS fingerprint spectra were determined and used to generate fingerprint maps. Each row in the figure represents all volatile flavor components in a given sample, and each column represents the abundance of each compound in different samples. Abundance differences are indicated by the color depth of the fingerprint spectra [47]. The deeper the red color, the higher the abundance of a substance, and the deeper the blue color, the lower the content of the abundance. After deodorization, new signals were present, so the diversity of volatile substances increased. GC-IMS detected 52 volatile substances, including monomers, dimers and a few trimers, including 22 aldehydes, 9 ketones, 14 alcohols, 4 pyrazines and 1 ester. All four samples were clearly different in the relative abundance and composition of volatile substances (Figure 12).

In the blue area, PJ deodorization markedly increased the concentration of volatile flavor compounds, with abundant aldehydes, including heptanal, nonanal, hexanal, pentanal, (E, E)-2,4-nonadienal, (E)-2-heptanal, 2-hexenal, (E,E)-2,4-heptadienal, 3-methylbutanal, (E)-2-pentenal and (E)-2-octenal, which appeared to result from transfer of aldehydes from the perilla juice, and the promotion of fat decomposition. Aldehydes are oxidation products of fatty acids and other organic compounds; these aldehydes are established indicators of quality degradation because of their low detection thresholds. Aldehydes such as hexanal, (E)-octenal, (E)-2-heptenal, (E,E)-2,4-heptadienal, heptanal and nonanal produce undesirable odor characteristics, including fishy, fatty, grassy and earthy/mold-like, whereas others produce pleasant aromas, such as clear, fruity and nutty. Aldehydes are recognized as primary contributors to the characteristic flavor profile of fish and fish products [48]. The oxidation process of polyunsaturated fatty acids generates various aldehydes, which contribute strongly to the flavor of aquatic products [49]. Jing et al. [50] determined that the main volatile compounds that cause the fishy odor of silver carp surimi are hexanal, nonanal and (E,E)-2,4-heptadienal. Some of the alcohols, ketones and pyrazines also increased in abundance, including 1-pentanol, 2-ethyl-1-hexanol, 2-ethyl-3-methylpyrazine, 2-ethyl-3,5-dimethylpyrazine, 2-heptanone and 2-butanone.

In the yellow area, 3-methylbutan-1-ol, ethyl acetate, 4-hydroxy-4-methyl-2-pentanone and 2,3-pentadione were most abundant after CW and PJ-CW deodorization. Alcohols are degradation products of lipids, particularly of polyunsaturated fatty acid oxidation [51]. The saturated alcohols, hexanol, pentanol and 3-methylbutanol have relatively high detection thresholds, so their contribution to the flavor is very small [9].

In the red area, CW deodorization resulted in slightly higher concentrations of hexanol and 2-pentanone compared with the other treatments. The aromatic components in CW, such as esters, aldehydes and ketones, should enhance the aroma of fish during cooking and mask or react with fishy odors, such as ammonia and hydrogen sulfide, thereby improving the overall flavor.

In the purple area, 3-hydroxy-2-butanone, benzaldehyde, benzenacetaldehyde and 5-methyl-2 (3H)-furanone were the most abundant after PJ-CW treatment. The presence of pyrazines in fish resulted from the conversion of amino acids, peptides and other nitrogen-containing compounds into pyrazine precursors. During deodorization, pyrazines are also formed by the conversion of precursors in the spices added to the deodorization solution, and through the Millard reaction [49].

## 4. Conclusions

In this study, deodorization by treatment with perilla juice (PJ), cooking wine (CW) and a mixture of the two (PJ-CW) were used successfully to reduce the undesirable fishy odor of Paddy Field Carp meat. E-nose analysis successfully distinguished between the odor characteristics of the untreated blank and the three deodorization treatments, the three deodorization treatments all reduced the content of sulfides; PJ-CW treatment resulted in the greatest reduction. The E-tongue analysis revealed an enhancement in the umami, bitterness, sourness and astringency attributes after deodorization; the umami-enhancing effect of the PJ-CW treatment was the greatest, followed by CW. Sweet and bitter amino acids were the most abundant of the four types of taste-active amino acids. CMP was the most abundant taste nucleotide in Paddy Field Carp, and the content of the nucleotides, AMP, CMP and IMP significantly increased. The equivalent umami concentration was the highest after PJ-CW deodorization, indicating that this treatment had the greatest synergistic effect between amino acids and nucleotides on the taste profile. GC-IMS analysis detected, identified and quantified fifty-two volatile flavor compounds, including twenty-two aldehydes, nine ketones, fourteen alcohols, four pyrazines and one ester. The abundance of volatile flavor substances after deodorization treatment significantly increased compared with the untreated blank. These findings provided a theoretical foundation for the study of flavor and taste differences under diverse deodorization conditions, ultimately leading to the identification of the optimal deodorization method for Paddy Field Carp.

## Figures and Tables

**Table 1:** Performance characteristics of PEN3 electronic nose sensor.

Sensor Serial Number	Sensor Name	Sensitive Substances
1	W1C	Aromatic constituents, benzene
2	W5S	Nitrogen oxides
3	W3C	Aromatic constituents, ammonia
4	W6S	Hydrides
5	W5C	Short-chain alkane aromatic component
6	W1S	Methyl
7	W1W	Sulfides
8	W2S	Alcohols, aldehydes and ketones
9	W2W	Aromatic ingredients, organic sulfides
10	W3S	Long-chain alkanes

**Table 2:** The contents, thresholds, taste attributes and TVA of free amino acids in the muscle of Paddy Field Carp with different deodorization treatments (mg/100 g).

Amino Acid	Taste Contribution	Thres-hold	B		PJ		CW		PJ-CW	
			Content mg/100 g	TAV	Content mg/100 g	TAV	Content mg/100 g	TAV	Content mg/100 g	TAV
Methionine (Met *)	bitter/sweet	30	245.73 ± 4.23 ^c^	8.19	284.36 ± 4.93 ^b^	9.48	282.66 ± 2.12 ^b^	9.42	411.58 ± 6.00 ^a^	13.72
Proline (Pro)	bitter/sweet	300	487.18 ± 10.20 ^c^	1.62	632.89 ± 26.58 ^ab^	2.11	600.97 ± 23.28 ^b^	2.00	662.85 ± 33.19 ^a^	2.21
Glycine (Gly)	sweet	130	714.40 ± 25.62 ^d^	5.50	941.21 ± 4.51 ^b^	7.24	810.93 ± 11.61 ^c^	6.24	980.45 ± 17.04 ^a^	7.54
Cysteine (Cys)	bitter/sweet	-	61.04 ± 1.62 ^c^	/	55.02 ± 0.25 ^d^	/	66.77 ± 0.58 ^b^	/	86.55 ± 1.95 ^a^	/
Glutamate (Glu)	umami /sour	30	78.21 ± 1.17 ^c^	2.61	66.97 ± 1.67 ^d^	2.23	85.66 ± 1.80 ^b^	2.86	95.36 ± 1.51 ^a^	3.18
Glutamine (Gln)	umami	-	50.03 ± 1.39 ^b^	/	42.22 ± 1.14 ^c^	/	49.59 ± 1.69 ^b^	/	55.00 ± 2.88 ^a^	/
Arginine (Arg)	bitter/sweet	50	1353.27 ± 21.61 ^c^	27.06	1491.16 ± 36.78 ^b^	29.82	1457.82 ± 21.61 ^b^	29.16	1642.33 ± 17.92 ^a^	32.85
1-Methyl-L-histidine (1-M-His)	-	-	9.25 ± 0.08 ^d^	/	10.08 ± 0.24 ^c^	/	11.57 ± 0.33 ^b^	/	12.48 ± 0.29 ^a^	/
3-Methyl-L-histidine (3-M-His)	-	-	10.69 ± 0.38 ^b^	/	11.75 ± 0.46 ^c^	/	12.42 ± 0.44 ^b^	/	15.05 ± 0.31 ^a^	/
Lysine (Lys *)	sweet/bitter	50	1480.14 ± 24.28 ^c^	29.60	1791.36 ± 26.00 ^b^	35.83	1737.50 ± 33.99 ^b^	34.75	1967.42 ± 34.61 ^a^	39.35
Tyrosine (Tyr)	bitter	-	285.57 ± 5.54 ^d^	/	330.87 ± 5.60 ^b^	/	316.68 ± 4.59 ^c^	/	410.23 ± 9.09 ^a^	/
Leucine (Leu *)	bitter	190	1120.53 ± 20.13 ^c^	5.90	995.71 ± 32.01 ^d^	5.24	1228.68 ± 22.11 ^b^	6.47	1367.03 ± 5.64 ^a^	7.19
hydroxyproline (Hy-Pro)	sweet	-	18.59 ± 0.50 ^d^	/	53.56 ± 1.65 ^a^	/	25.03 ± 0.96 ^c^	/	31.39 ± 0.84 ^b^	/
Threonine (Thr *)	sweet	260	740.87 ± 19.44 ^b^	2.85	746.55 ± 10.96 ^b^	2.87	760.99 ± 26.89 ^b^	2.93	899.33 ± 12.91 ^a^	3.46
Serine (Ser)	sweet	150	538.58 ± 19.08 ^b^	3.59	559.45 ± 12.93 ^b^	3.73	614.20 ± 21.74 ^a^	4.09	573.96 ± 22.11 ^b^	3.83
Valine (Val *)	sweet/bitter	40	573.61 ± 18.85 ^b^	14.34	555.41 ± 17.84 ^b^	13.88	552.15 ± 17.36 ^b^	13.80	657.49 ± 16.26 ^a^	16.44
Isoleucine (Ile *)	bitter	90	628.23 ± 19.74 ^c^	6.98	603.72 ± 19.84 ^c^	6.71	694.56 ± 10.73 ^b^	7.72	753.31 ± 24.48 ^a^	8.37
Histidine (His)	bitter/sour	20	381.74 ± 17.59 ^b^	19.09	373.21 ± 14.28 ^b^	18.66	356.01 ± 4.77 ^b^	17.80	504.17 ± 13.52 ^a^	25.21
SWAA			6213.41 ± 44.68 ^d^		7110.97 ± 99.30 ^b^		6909.02 ± 69.18 ^c^		7913.34 ± 55.86 ^a^	
BIAA			6617.04 ± 33.32 ^d^		7113.72 ± 116.96 ^c^		7293.80 ± 45.00 ^b^		8462.95 ± 92.17 ^a^	
UMAA			128.24 ± 2.21 ^c^		109.20 ± 1.22 ^d^		135.25 ± 2.47 ^b^		150.37 ± 3.87 ^a^	
SOAA			459.95 ± 17.21 ^b^		440.18 ± 15.18 ^b^		441.67 ± 6.42 ^b^		599.53 ± 14.56 ^a^	
EAA			4789.11 ± 35.40 ^d^		4977.11 ± 83.51 ^c^		5256.55 ± 40.40 ^b^		6056.16 ± 40.99 ^a^	
TFAA			8777.67 ± 41.94 ^c^		9545.51 ± 125.65 ^b^		9664.19 ± 91.11 ^b^		11,125.98 ± 92.68 ^a^	

Note: B: blank group; PJ: perilla juice; CW: cooking wine; PJ-CW: perilla juice and cooking wine. TAV: taste activity value. “-”: did not find data; “/”: was not calculated; “*”: essential amino acids. “SWAA” is sweet amino acid; “BIAA” is bitter amino acid; “UMAA” is umami amino acid; “SOAA” is sour amino acid; “EAA” is essential amino acid; “TFAA” is free amino acid; the same line marking different letters indicates significant differences (*p* < 0.05).

**Table 3:** The contents, thresholds, EUC and TVA of nucleotide in muscle of Paddy Field Carp with different deodorization treatments.

Amino Acid	Threshold (mg/100 g)	B		PJ		CW		PJ-CW	
		Content (mg/100 g)	TAV	Content (mg/100 g)	TAV	Content (mg/100 g)	TAV	Content (mg/100 g)	TAV
CMP	-	36.89 ± 0.53 ^d^	/	59.00 ± 0.46 ^c^	/	84.89 ± 0.86 ^a^	/	81.90 ± 1.23 ^b^	/
AMP	50	4.21 ± 0.03 ^d^	0.08	4.46 ± 0.04 ^c^	0.09	4.96 ± 0.05 ^b^	0.10	5.87 ± 0.03 ^a^	0.12
IMP	25	1.35 ± 0.03 ^c^	0.05	1.05 ± 0.01 ^d^	0.04	1.56 ± 0.04 ^a^	0.06	1.45 ± 0.03 ^b^	0.06
EUC (gMSG/100 g)		2.64		2.32		3.07		3.15	

Note: B: blank group; PJ: perilla juice; CW: cooking wine; PJ-CW: perilla juice and cooking wine. “AMP” is 5′-adenosine monophosphate; “GMP” is 5′-guanosine monophosphate, and “CMP” is 5′-cytidine monophosphate. “TAV”: taste activity value. Different superscript letters in the same row indicate significant differences (*p* < 0.05).

## Data Availability

The original contributions presented in the study are included in the article, further inquiries can be directed to the corresponding author.
